# Anti-Double-Stranded DNA Isotypes and Anti-C1q Antibody Improve the Diagnostic Specificity of Systemic Lupus Erythematosus

**DOI:** 10.1155/2018/4528547

**Published:** 2018-09-27

**Authors:** Yu Jia, Lingling Zhao, Chunyan Wang, Jin Shang, Yi Miao, Yangyang Dong, Zhanzheng Zhao

**Affiliations:** ^1^Clinical Laboratory of Nephrology, First Affiliated Hospital of Zhengzhou University, Zhengzhou 450052, China; ^2^Blood Transfusion Department, Shanghai Xuhui Central Hospital, Shanghai 200031, China; ^3^Department of Nephrology, First Affiliated Hospital of Zhengzhou University, Zhengzhou 450052, China

## Abstract

**Objectives:**

We aimed to evaluate the value of immunoglobulin (Ig) G, IgM, and IgA isotypes of anti-double-stranded DNA (anti-dsDNA) and anti-C1q antibody in diagnosing systemic lupus erythematosus (SLE) patients and elucidate their association with disease activity and lupus nephritis.

**Methods:**

Blood samples were obtained from 96 SLE patients, 62 other autoimmune disease patients, and 60 healthy blood donors. Anti-dsDNA IgG, IgM, and IgA isotypes and anti-C1q antibody were measured by enzyme-linked immunosorbent assay. Disease activity of SLE patients was assessed according to the SLE Disease Activity Index score.

**Results:**

When specificity was greater than 90%, the sensitivity of anti-dsDNA IgG, IgM, and IgA isotypes and anti-C1q antibody in diagnosing SLE was 75%, 45%, 33%, and 49%, respectively. The prevalence of anti-dsDNA IgG (*p* = 0.002), anti-dsDNA IgA (*p* = 0.028), and anti-C1q antibody (*p* = 0.000) in active cases was significantly higher than those in inactive ones. In addition, the presence of anti-C1q antibody was associated with renal involvement (*p* = 0.032). Anti-dsDNA IgM showed no significant association with disease activity, but it was inversely linked with lupus nephritis (*p* = 0.005). When anti-dsDNA IgG and IgA and anti-C1q were combined to evaluate SLE disease activity, the specificity reached the highest level (90%). When anti-C1q positive was accompanied by anti-dsDNA IgM negative, the specificity of diagnosing lupus nephritis was up to 96%.

**Conclusions:**

This study demonstrated the role of anti-dsDNA IgG, IgM, and IgA isotypes and anti-C1q antibody alone or combination in diagnosing SLE. Anti-dsDNA IgG and IgA and anti-C1q were shown to be associated with disease activity, while anti-dsDNA IgM and anti-C1q were associated with lupus nephritis. When the related antibodies were combined, the diagnostic specificity was significantly higher.

## 1. Introduction

Systemic lupus erythematosus (SLE) is an autoimmune disease characterized by the presence of diverse autoantibodies and damage to multiple tissues and organs. Lupus nephritis (LN) is one of the most serious complications of SLE, and it is associated with increased morbidity and mortality. The diagnosis of SLE and LN at the very early stage and achieving a correct assessment of disease activity remain great challenges due to the clinical and serological heterogeneity [1–3].

Anti-double-stranded DNA (anti-dsDNA) antibodies have been established as one of the American College of Rheumatology (ACR) and Systemic Lupus International Collaborating Clinics' criteria for the diagnosis of SLE [4–6]. Detectable levels of anti-dsDNA immunoglobulin (Ig) G precede clinical diagnosis by at least 2 years [7, 8], and its concentration fluctuates with disease activity of SLE [9, 10]. However, it has been demonstrated that anti-dsDNA IgM does not correlate with disease activity, and evidence from mouse model showed that it might have a protective role against the development of LN [11–14]. With respect to anti-dsDNA IgA, the results are controversial regarding whether it is linked to active disease, LN, or both [15–18]. In spite of the prevalence of anti-dsDNA IgM and IgA isotypes in SLE, they are not routinely measured during the follow-up of these patients, and their diagnostic values in SLE are still a topic of considerable interest.

In addition to anti-dsDNA autoantibodies, anti-C1q antibody has been suggested to exhibit a pathogenic importance in SLE and LN. C1q is the first component of the classical complement pathway, playing an important role in the clearance of immune complexes from tissues and apoptotic cell debris [19–21]. Hereditary deficiency of C1q is the strongest genetic risk factor for the development of SLE, about 90% of patients with homozygous C1q deficiency developing SLE or lupus-like syndromes. However, this genetic deficiency is rare. Instead, SLE patients often have secondary C1q deficiency caused by the presence of anti-C1q antibody [22–24]. Anti-C1q antibody was first reported in the serum of SLE patients in 1984, and its prevalence ranges from 34% to 47% [25–27]. A multitude of studies have been performed to identify the correlation between serum anti-C1q antibody levels and SLE. It has been shown that anti-C1q antibody might be a predictor of proliferative LN and is more closely correlated with renal disease activity than other autoantibodies like anti-dsDNA [22, 28–31]. Even so, the diagnostic value of anti-C1q antibody in SLE and LN remains debatable and more research is needed.

The combination of anti-C1q antibody and anti-dsDNA IgG was reported to have a stronger serological association with renal involvement [32]. However, the diagnostic significance of anti-C1q in combination with other subtypes of anti-dsDNA in SLE has not been demonstrated. Given that anti-dsDNA IgG, IgM, and IgA isotypes may be produced in different periods of the disease and reflect different disease states, it is particularly important to use these biomarkers rationally and to combine them with other important biological indicators, such as anti-C1q, at the same time in order to more accurately diagnose diseases. As a result, optimized permutation and combination of anti-dsDNA isotypes and anti-C1q should be further explored to find the best diagnostic strategy that will contribute to clinical comprehensive diagnosis in a beneficial manner.

Thus, the objective of the current study was to evaluate the role of anti-dsDNA IgG, IgM, and IgA isotypes as well as anti-C1q antibody alone or in combination with the isotypes in the diagnosis of SLE and to elucidate their association with disease activity and LN.

## 2. Materials and Methods

### 2.1. Ethics Statement

The study was approved by the ethics committee at the First Affiliated Hospital of Zhengzhou University (Zhengzhou, China), and written informed consent was obtained from every participant. For those patients involved in the study who were younger than 18 years, written informed consent was obtained from their guardians or parents.

### 2.2. Patients

The sera of 96 consecutive SLE patients diagnosed according to ACR criteria [32] were collected from the First Affiliated Hospital of Zhengzhou University from July 2016 to May 2017. At the beginning of the study, the 96 SLE patients presented a mean ± standard deviation age of 33.54 ± 12.93 years (range: 9–70 years), a median duration of disease of 20 months (range: 0–216 months), and a female to male ratio of 7 : 1. Clinical features and laboratory findings of 96 SLE patients are shown in Table 1. Among them, 7 individuals did not undergo complement testing, and 2 patients had Sjogren syndrome. In addition, the sera of 62 patients suffering from other autoimmune diseases were collected, including sera from 16 patients with systemic vasculitis, 9 with autoimmune hepatitis, 9 with primary antiphospholipid syndrome, 8 with Sjögren's syndrome, 8 with connective tissue disease, 7 with rheumatoid arthritis, 3 with systemic sclerosis, 1 with ankylosing spondylitis, and 1 with Guillain–Barré syndrome. Sera from 60 healthy blood donors were also included. All of the sera were frozen at −20°C until they were processed.

The SLE Disease Activity Index (SLEDAI) was used for the assessment of disease activity. A patient with SLEDAI ≥ 10 was defined as having active SLE [33–35]. The diagnosis of LN relied on urinalysis, renal functions, and kidney biopsies. Kidney biopsies of LN patients were classified according to the International Society of Nephrology-Renal Pathology Society (ISN/RPS) classification by an independent pathologist. Active LN was defined as urine protein excretion ≥500 mg/day or cellular casts [4].

### 2.3. Detection of Anti-dsDNA and Anti-C1q Antibodies by ELISA

Anti-dsDNA IgG, IgM, and IgA isotypes and anti-C1q antibodies were measured using enzyme-linked immunosorbent assay (ELISA) kits (Orgentec Diagnostika GmbH, Mainz, Germany) on the automatic ELISA reader Alegria® (Orgentec Diagnostika GmbH, Mainz, Germany) according to the manufacturer's instructions. Autoantibodies were determined on the Alegria® test strips, based on human recombinant dsDNA or highly purified human C1q as the antigen bound to the microwells. Positivity cutoffs were set at ≥20 U/ml for anti-dsDNA isotypes and ≥10 U/ml for anti-C1q antibodies, respectively, in compliance with the manufacturer's instructions.

### 2.4. Statistical Analysis

Statistical analyses were carried out using software SPSS for Windows (version 16.0; IBM Inc., New York, USA). The association between qualitative variables was evaluated by a chi-squared test, and the quantitative variables were compared by *t*-test or Mann–Whitney *U* test. Data are presented in the format of mean ± standard deviation or mean ± standard error of mean (SEM). All of the tests were used with two-sided options, and the significance level was set at a *p* value of 0.05.

## 3. Results

### 3.1. Positive Rate of Serum Anti-dsDNA Isotypes and Anti-C1q Antibody in SLE Patients, Other Autoimmune Disease Patients, and Healthy Controls

In the cohort of 96 SLE patients, 68 cases (70.8%) were anti-dsDNA IgG positive, 43 cases (44.8%) were anti-dsDNA IgA positive, 32 cases (33.3%) were anti-dsDNA IgM positive, and 46 cases (47.9%) were anti-C1q antibody positive. In the non-SLE autoimmune diseases group, 8 cases (12.9%) were anti-dsDNA IgG isotype positive, 2 cases (3.2%) were IgA positive, 2 cases (3.2%) were IgM positive, and 8 cases (12.9%) were anti-C1q antibody positive. In healthy donors, positive cases of anti-dsDNA IgG, IgA, IgM, and anti-C1q antibodies were 1 (1.7%), 2 (3.3%), 2 (3.3%), and 2 (3.3%), respectively (Table 2).

### 3.2. Associations of the Presence of Anti-dsDNA Isotypes and Anti-C1q Antibody with Active/Inactive SLE Patients, LN/Non-LN Groups, and Active/Inactive LN Cases

For the 96 SLE patients, disease activity was evaluated according to the SLEDAI score. In cases of active disease (SLEDAI ≥ 10), the prevalence of anti-dsDNA IgG (47/57, 82.5% vs. 21/39, 53.8%, *p* = 0.002), anti-dsDNA IgA (24/57, 42.1% vs. 8/39, 20.5%, *p* = 0.028), and anti-C1q (36/47, 63.2 vs. 10/39, 25.6%, *p* = 0.000) was significantly higher than in the inactive cases. In contrast, there was no significant difference in the prevalence of anti-dsDNA IgM in the active versus the inactive SLE patients (Table 3).

In 50 LN patients, 4 did not have pathological examination of nephropathy, and 6 had pathological tests in other hospitals. In the other 40 LN patients, we tested immunoglobulins (IgG, IgM, and IgA), C3, C4, and C1q in glomeruli due to the direct immunofluorescence, of which both IgG and IgM were positive in all patients. In addition, we found the presence of anti-dsDNA IgG in sera was correlated with the deposits of C3 (*p* = 0.004), C4 (*p* = 0.034), and C1q (*p* = 0.043) in glomeruli; anti-dsDNA IgA in sera was associated with IgA (*p* = 0.026) deposited in glomeruli (Supplement Table 1).

When we compared the positivity of studied antibodies between the groups of patients with and without LN, we found that the presence of anti-C1q antibody (28/50, 56% vs. 18/46, 39.1%, *p* = 0.032) was correlated with renal involvement. However, anti-dsDNA IgG or anti-dsDNA IgA showed no correlation. Furthermore, a pronounced negative association of anti-dsDNA IgM isotype with LN (*p* = 0.005) was demonstrated. Of the 50 SLE patients with LN, only 16 (32%) exhibited anti-dsDNA IgM positivity in comparison with 27 (58.7%) of the 46 patients without LN who exhibited such (Table 3).

The associations of anti-dsDNA isotypes and anti-C1q antibody with active LN and/or inactive LN did not exist as shown in Table 3.

### 3.3. Levels of Serum Anti-dsDNA Isotypes and Anti-C1q Antibody in Active, Inactive, LN, Non-LN SLE Patients and Active, Inactive LN Patients

As shown in Table 4, the mean levels of anti-dsDNA IgG (116.66 U/ml ± 10.63 U/ml vs. 62.21 U/ml ± 11.27 U/ml, *p* = 0.001), anti-dsDNA IgA (44.39 U/ml ± 8.03 U/ml vs. 17.89 U/ml ± 5.61 U/ml, *p* = 0.003), and anti-C1q (29.36 U/ml ± 4.17 U/ml vs. 11.44 U/ml ± 2.79 U/ml *p* = 0.000) were significantly higher in active SLE patients versus those in inactive SLE patients. Anti-dsDNA IgM isotype (26.14 U/ml ± 5.72 U/ml vs. 45.80 U/ml ± 8.16 U/ml, *p* = 0.037) was significantly lower in LN patients than that in non-LN patients. However, the levels of these autoantibodies could not distinguish active LN from inactive LN.

### 3.4. Diagnostic Value of Anti-dsDNA Isotypes, Anti-C1q Antibody, and Low C3 and/or C4 in SLE Patients

Sensitivity and specificity of the anti-dsDNA IgG class (75% and 93%) for the diagnosis of SLE were superior to those for anti-C1q antibody (49% and 92%). The anti-dsDNA IgM class as well as the IgA class showed less sensitivity (45% and 33%, respectively) but high specificity (97% and 97%, respectively). Low C3 and/or C4 (81% sensitivity and 59% specificity) was more sensitive than anti-dsDNA isotypes and anti-C1q antibody for the diagnosis of SLE, but it showed significantly lower specificity than them. Positive predictive value (PPV), negative predictive value (NPV), and odds ratio (OR) for SLE diagnosis were 88%, 84%, and 37.667 for anti-dsDNA IgG class, 92%, 69%, and 23.731 for IgM, 89%, 65%, and 14.625 for IgA, 58%, 83%, and 10.679 for anti-C1q antibody, and 62%, 79%, and 6.097 for low C3 and/or C4 (Table 5).

### 3.5. Diagnostic Value of Anti-dsDNA Isotypes, Anti-C1q Antibody, and Low C3 and/or C4 in Disease Activity of SLE Patients

In Table 6, we show the analysis results of the sensitivity, specificity, PPV, NPV, and OR of anti-dsDNA IgG, anti-dsDNA IgA, and anti-C1q antibodies alone or in combination for identification of patients with active SLE. When calculated alone, anti-dsDNA IgG turned out to demonstrate the highest sensitivity and lowest specificity (83% and 46%), whereas anti-dsDNA IgA displayed the lowest sensitivity and highest specificity (42% and 80%). The anti-C1q antibody showed moderate sensitivity and specificity (63% and 74%). Combinations of each antibody could significantly increase the specificity but decrease the sensitivity for identifying patients with active SLE in comparison with these antibodies alone. In particular, when anti-dsDNA IgG, anti-dsDNA IgA, and anti-C1q antibodies were all positive, the specificity was as high as 90%, in spite of the low sensitivity (32%). In addition, sensitivity and specificity of the low C3 and/or C4 for the diagnosis of active SLE were 80% and 71%, respectively.

### 3.6. Diagnostic Value of Anti-dsDNA Isotypes and Anti-c1q Antibody in Lupus Nephritis Patients

As mentioned above, the presence of anti-C1q antibody and the absence of anti-dsDNA IgM class were associated with LN. In Table 7, we show the diagnostic value for renal involvement of both antibodies performed. For the diagnosis of LN, sensitivity, specificity, PPV, NPV, and OR of anti-C1q antibody were 59%, 63%, 64%, 58%, and 2.407; additionally, those of anti-dsDNA IgM were 70%, 59%, 69%, 64%, and 3.316, respectively. Of note, when anti-C1q antibody was positive and anti-dsDNA IgM was negative simultaneously, the specificity (96%) was high with 34% sensitivity, 90% PPV, 57% NPV, and 11.333 OR (Table 7).

## 4. Discussion

In the present study, we investigated the diagnostic value of anti-dsDNA IgG, IgM, and IgA isotypes and anti-C1q antibody in SLE patients. The results indicated that all of the antibodies considered were highly specific for the diagnosis of SLE, although anti-dsDNA IgM, IgA, and anti-C1q antibody were less sensitive than anti-dsDNA IgG.

In agreement with the results of a previous study [36], we found that anti-dsDNA IgG antibody was associated with the disease activity of SLE. The prevalence and concentrations of IgG class were significantly higher in active cases than that in inactive ones. In addition, anti-dsDNA IgG was considered to be one of multiple autoantibodies implicated in the pathogenesis of LN which was proved by a previous animal study [36]. However, we were not able to demonstrate a significant association of anti-dsDNA IgG with kidney involvement. The positivities of anti-dsDNA IgG in groups with or without kidney involvement were both high at 76% and 71.7%, respectively. The results were similar to those noted by Villalta et al. and Atta et al. [15, 16].

The role of anti-dsDNA IgA antibody in diagnosing and monitoring SLE is rarely reported, and the available results are conflicting. Villalta et al. and Miltenburg et al. [15, 18] observed that anti-dsDNA IgA was linked with SLE disease activity and LN, whereas the studies of Witte et al. and Atta et al. [17, 16] suggested a lack of association between IgA class and LN. In our study, anti-dsDNA IgA was shown to be a risk factor for active disease in SLE but not for LN. In addition, anti-dsDNA IgA was supposed to be associated with joint abnormalities [18] or vasculitis [11], but we found it may be correlated with serositis (*p* = 0.008) and anemia (*p* = 0.004) (Supplement Table 2). Nevertheless, these correlations need to be verified in large cohorts of SLE patients.

This disagreement about the diagnostic value of IgG and IgA anti-dsDNA isotypes in SLE patients among different researchers may be caused by the use of different detection methods, different reagent manufactures, different genetic backgrounds of the populations, or the different courses of patients involved in these studies. What is more, it has been reported that different subclasses of IgG anti-dsDNA are not equally pathogenic. In fact, IgG1 and IgG3 are the major pathogenic subtypes, while IgG2 and IgG4 are not [37]. Controversy pathogenicity of different anti-dsDNA IgG subclasses in SLE patients may cause the diagnostic discrepancy of the positive test result.

Anti-dsDNA IgM antibody appears to play a protective role in the development of LN, and this concept has been verified previously in mouse models: specifically, mice treated with IgM anti-dsDNA exhibited attenuated renal pathology and improved survival [12]. Our research also revealed a negative correlation between IgM class and LN. Of the 43 IgM-positive SLE patients, only 37.2% (16/43) presented LN, which is significantly less than non-LN cases (62.8%, 27/43). Meanwhile, in line with other studies, we found that anti-dsDNA IgM could not be a significant parameter to distinguish patients with active disease from inactive diseases.

Intriguingly, it is noteworthy that, among the autoantibodies we examined in the current study, anti-C1q antibody was the only biomarker associated with both disease activity and LN. In active SLE patients, the positivity and average titre of anti-C1q were significantly higher than those in inactive patients. Likewise, as compared with patients without renal involvement, the prevalence of anti-C1q in patients with LN was significantly higher, although the mean concentration did not differ between the two groups. These results suggest that anti-C1q antibody may be a potential and useful serological biomarker even superior to anti-dsDNA in evaluating the disease activity of SLE and LN.

However, no significant correlations were found between all of these autoantibodies and the activity of LN in this study.

Low C3 and/or C4 are known as important biomarkers for SLE. Our study indicated that low C3 and/or C4 were more sensitive but of significantly lower specificity than anti-dsDNA isotypes and anti-C1q antibody for the diagnosis of SLE. For the diagnosis of active SLE, anti-dsDNA IgG had slightly higher sensitivity than low C3 and/or C4 although its specificity was lower; while anti-C1q antibody and anti-dsDNA IgA had slightly higher specificity than low C3 and/or C4 although they had lower sensitivities.

Indeed, both anti-dsDNA antibodies and anti-C1q antibody play indispensable roles in the pathogenesis of SLE. The deposition of immune complexes containing pathogenic anti-dsDNA antibodies in glomeruli initiate LN, but that is far from sufficient: notably, classical complement activation is another important mechanism, and administering anti-C1q antibodies to mice exacerbates glomerular immunoglobulin deposition. Thus, we supposed the combined measurement of anti-dsDNA isotypes and anti-C1q antibody might enhance the efficiency of estimating disease activity and kidney involvement. Our results showed that, in the evaluation of SLE disease activity, when anti-dsDNA IgG, IgA, and anti-C1q were combined, the specificity reached its highest (90%) with 32% sensitivity and the corresponding positive predictive value was also up to 82%. Similarly, for the diagnosis of LN, when an anti-C1q-positive finding was accompanied by an anti-dsDNA IgM-negative finding, the specificity was up to 96% in spite of 34% sensitivity, and the positive predictive value was 90%. In other words, the combined detection of different antibodies significantly increased the specificities in comparison with the detection of these biomarkers alone, providing clinicians with a more accurate diagnosis quickly.

In conclusion, our study confirmed the diagnostic value of anti-dsDNA IgG, IgM, and IgA isotypes and anti-C1q antibody in SLE patients. Anti-dsDNA IgG, IgA, and anti-C1q can be used to evaluate disease activity, and anti-dsDNA IgM as well as anti-C1q can help to better distinguish between LN and non-LN patients. Furthermore, a combination of related antibodies can obviously increase the specificity of diagnosis. Additional studies should be performed involving larger cohorts of SLE patients, and longitudinal research will be necessary to evaluate the ability of each antibody to assess the prognosis of the disease and the therapeutic effect.

## Figures and Tables

**Table 1:** Clinical features and laboratory findings in 96 SLE patients.

Clinical characteristics		
Sex (M/F)	12/84	
Disease duration median (range) in months	20 (0–216)	
SLEDAI score median (range)	10 (0–30)	
*Organ involvement*	Number	%
Central nervous system involvement	5	5.2
Skin rashes	35	36.5
Arthritis	8	8.3
Serositis	39	40.6
Vasculitis	15	15.6
Anemia	39	40.6
Leukopenia	28	29.2
Thrombocytopenia	27	28.1
*Autoantibody profile*		
ANA positivity	96	100
Anti-Sm	34	35.4
Anti-U1RNP	38	39.6
Anti-SSA	57	69.8
Anti-SSB	17	17.7
IgG anti-dsDNA	68	70.8
IgM anti-dsDNA	43	44.8
IgA anti-dsDNA	32	33.3
Anti-C1q	28	29.2
*Complement levels*		
Low C3	43/89	48.3
Low C4	44/89	49.4

M: male; F: female; SLEDAI: SLE disease activity index; ANA: antinuclear antibodies; anti-Sm: anti-Smith; anti-dsDNA: anti-double-stranded DNA.

**Table 2:** A comparison of the group of SLE patients to a group of other autoimmune diseases and healthy controls.

	SLE	Other autoimmune diseases	Healthy controls
Anti-dsDNA IgG	70.8% (68/96)	12.9% (8/62)	1.7% (1/60)
Anti-dsDNA IgM	44.8% (43/96)	3.2% (2/62)	3.3% (2/60)
Anti-dsDNA IgA	33.3% (32/96)	3.2% (2/62)	3.3% (2/60)
Anti-C1q	47.9% (46/96)	12.9% (8/62)	3.3% (2/60)

*SLE*: systemic lupus erythematosus.

**Table 3:** Associations of the presence of anti-dsDNA isotypes and anti-C1q antibody with active/inactive SLE patients, LN/non-LN groups, and active/inactive LN cases.

	Activity of SLE (*N* = 96)			SLE with renal involvement (*N* = 96)			Activity of LN (*N* = 50)		
	Active SLE	Inactive SLE	*p* value	LN	Non-LN	*p* value	Active LN	Inactive LN	*p* value
Anti-dsDNA IgG	82.5% (47/57)	53.8% (21/39)	0.002	76% (38/50)	71.7% (33/46)	NS	78.8% (26/33)	58.8% (10/17)	NS
Anti-dsDNA IgM	49.1% (28/57)	38.5% (15/39)	NS	32% (16/50)	58.7% (27/46)	0.005	33.3% (11/33)	29.4% (5/17)	NS
Anti-dsDNA IgA	42.1% (24/57)	20.5% (8/39)	0.028	28% (14/50)	37.0% (17/46)	NS	30.3% (10/33)	29.4% (5/17)	NS
Anti-C1q	63.2% (36/57)	25.6% (10/39)	0.000	56% (28/50)	39.1% (18/46)	0.032	33.3% (11/33)	23.5% (4/17)	NS

SLE: systemic lupus erythematosus; LN: lupus nephritis; NS: no significance; *p* < 0.05, chi-squared test.

**Table 4:** Concentrations (mean ± SEM, U/ml) of anti-dsDNA isotypes and anti-C1q antibody in active, inactive, LN, and non-LN SLE patients and active, inactive LN patients.

	Activity of SLE (*N* = 96)			SLE with renal involvement (*N* = 96)			Activity of LN (*N* = 50)		
	Active SLE (U/ml)	Inactive SLE (U/ml)	*p* value	LN (U/ml)	Non-LN (U/ml)	*P* value	Active LN (U/ml)	Inactive LN (U/ml)	*p* value
Anti-dsDNA IgG	116.66 ± 10.63	62.21 ± 11.27	0.001	95.57 ± 11.29	95.60 ± 12.17	NS	98.60 ± 13.40	88.90 ± 22.06	NS
Anti-dsDNA IgM	36.49 ± 5.99	34.20 ± 8.71	NS	26.14 ± 5.72	45.80 ± 8.16	0.037	26.61 ± 6.28	25.23 ± 11.90	NS
Anti-dsDNA IgA	44.39 ± 8.03	17.89 ± 5.61	0.003	29.45 ± 6.90	38.17 ± 8.53	NS	32.04 ± 9.44	23.13 ± 8.47	NS
Anti-C1q	29.36 ± 4.17	11.44 ± 2.79	0.000	23.07 ± 4.00	21.02 ± 4.12	NS	26.38 ± 5.52	16.62 ± 4.63	NS

SLE: systemic lupus erythematosus; LN: lupus nephritis; NS: no significance; *p* < 0.05, Mann–Whitney *U* test.

**Table 5:** Diagnostic value of anti-dsDNA isotypes, anti-C1q antibody, and low C3 and/or C4 for SLE.

	Sensitivity	Specificity	PPV	NPV	OR (95% CI)
Anti-dsDNA IgG	75%	93%	88%	84%	37.667 (16.270–87.204)
Anti-dsDNA IgM	45%	97%	92%	69%	23.731 (8.102–69.512)
Anti-dsDNA IgA	33%	97%	89%	65%	14.625 (4.951–43.203)
Anti-C1q	49%	92%	58%	83%	10.679 (4.917–23.193)
Low C3 and/or C4	81%	59%	62%	79%	6.097 (2.891–12.858)

SLE: systemic lupus erythematosus; PPV: positive predictive value; NPV: negative predictive value; OR: odds ratio; CI: confidence interval.

**Table 6:** Diagnostic value of anti-dsDNA isotypes, anti-C1q antibody, and low C3 and/or C4 for disease activity of SLE.

	Sensitivity	Specificity	PPV	NPV	OR (95% CI)
Anti-dsDNA IgG	83%	46%	69%	64%	4.029 (1.592–10.196)
Anti-dsDNA IgA	42%	80%	75%	48%	2.818 (1.103–7.203)
Anti-C1q	63%	74%	78%	58%	4.971 (2.025–12.202)
Anti-dsDNA IgG and IgA	42%	82%	77%	49%	3.325 (1.257–8.790)
Anti-dsDNA IgG and anti-C1q	54%	79%	79%	54%	8.495 (3.436–21.002)
Anti-dsDNA IgA and anti-C1q	32%	87%	78%	47%	3.138 (1.053–9.356)
Anti-dsDNA IgG, IgA, and anti-C1q	32%	90%	82%	47%	4.038 (1.246–13.085)
Low C3 and/or C4	80%	71%	81%	69%	9.6 (3.566–25.844)

SLE: systemic lupus erythematosus; PPV: positive predictive value; NPV: negative predictive value; OR: odds ratio; CI: confidence interval.

**Table 7:** Diagnostic value of anti-dsDNA isotypes and anti-c1q antibody for lupus nephritis.

	Sensitivity	Specificity	PPV	NPV	OR (95% CI)
Anti-C1q	59%	63%	64%	58%	2.407 (1.001–5.793)
Anti-dsDNA IgM negative	70%	59%	69%	64%	3.316 (1.427–7.702)
Anti-C1q positive and anti-dsDNA IgM negative	34%	96%	90%	57%	11.333 (2.446–52.502)

SLE: systemic lupus erythematosus; PPV: positive predictive value; NPV: negative predictive value; OR: odds ratio; CI: confidence interval.

## Data Availability

The statistical data used to support the findings of this study are available from the corresponding author upon request.

## Supplementary Materials

Supplementary materials Supplement Table 1: frequency of direct immunofluorescence results in the presence or absence of anti-DNA antibody isotypes and anti-C1q antibody in SLE-LN patients. Supplement Table 2: frequency of SLE clinical manifestations in the presence or absence of anti-dsDNA antibody isotypes and anti-C1q antibody (*N* = 96).
