# Supplementary material for: Anti-Double-Stranded DNA Isotypes and Anti-C1q Antibody Improve the Diagnostic Specificity of Systemic Lupus Erythematosus
Source: Dis Markers. 2018 Sep 27;2018:4528547. doi: 10.1155/2018/4528547 (PMC6180986; doi:10.1155/2018/4528547)
Supplement: Supplementary materials — Supplement Table 1: frequency of direct immunofluorescence results in the presence or absence of anti-DNA antibody isotypes and anti-C1q antibody in SLE-LN patients. Supplement Table 2: frequency of SLE clinical manifestations in the presence or absence of anti-dsDNA antibody isotypes and anti-C1q antibody (N = 96). [file 4528547.f1.pdf]

Supplement Table 1. Frequency of direct immunofluorescence results in presence or absence of anti-DNA antibody isotypes and anti-C1q antiboby in SLE-LN patients.

Direct immunofluorescence assay	***IgG- anti-dsDNA antibodies***			***IgM- anti-dsDNA antibodies***			***IgA- anti-dsDNA antibodies***			***Anti-C1q antibodies***		
	Positive Result (%)	***Negative Result (%)***	***p value****	***Positive Result (%)***	***Negative Result (%)***	***p value****	***Positive Result (%)***	***Negative Result (%)***	***p value****	***Positive Result (%)***	***Negative Result (%)***	***p value****
IgA	65.0	12.5	ns	30.0	47.5	ns	30.0	47.5	0.026	32.5	45	ns
C3	80.0	15.0	0.004	32.5	62.5	ns	30.0	65.0	ns	35.0	60	ns
C4	60.5	7.9	0.034	23.7	44.7	ns	23.7	44.7	ns	26.3	42.1	ns
C1q	80.0	17.5	0.043	32.5	65.0	ns	30.0	67.5	ns	35.0	62.5	ns

p* = Chi-square-test, ns = not significant.

Supplement Table 2. Frequency of SLE clinical manifestations in presence or absence of anti-dsDNA antibody isotypes and anti-C1q antiboby (N=96) .

	IgG- anti-dsDNA antibodies			***IgM- anti-dsDNA antibodies***			***IgA- anti-dsDNA antibodies***			***Anti-C1q antibody***		
Clinical variable	***Positive Result (%)***	***Negative Result (%)***	***p value****	***Positive Result (%)***	***Negative Result (%)***	***p value****	***Positive Result (%)***	***Negative Result (%)***	***p value****	***Positive Result (%)***	***Negative Result (%)***	***p value****
Central nervous system involvement (n=6)	4.2	2.1	ns	4.2	2.1	ns	3.1	3.1	ns	1.0	5.2	ns
Skin rashes (n=35)	25	11.5	ns	18.8	17.7	ns	16.7	19.8	ns	12.5	24.0	ns
Arthritis (n=8)	6.3	2.1	ns	5.2	3.1	ns	2.1	6.3	ns	3.1	5.2	ns
Serositis (n=39)	29.2	11.5	0.027	20.8	19.8	ns	19.8	20.8	0.008	15.6	25	ns
Hematological manifestations (n=15)	14.6	1.0	0.037	6.3	9.4	ns	5.2	10.4	ns	8.3	7.3	0.025
Anemia (n=27)	22.9	5.2	ns	15.6	12.9	ns	15.6	12.9	0.004	13.5	14.6	0.017
Leukopenia (n=27)	21.9	6.3	ns	15.6	12.5	ns	12.5	15.6	ns	11.5	16.7	ns
Thrombocytopenia (n=27)	21.9	6.5	ns	13.5	14.6	ns	10.8	17.7	ns	11.5	16.7	ns

p* = Chi-square-test, ns = not significant.
